# Advances in Wettability-Engineered Open Planar-Surface Droplet Manipulation

**DOI:** 10.3390/mi16080893

**Published:** 2025-07-31

**Authors:** Ge Chen, Jin Yan, Junjie Liang, Jiajia Zheng, Jinpeng Wang, Hongchen Pang, Xianzhang Wang, Zihao Weng, Wei Wang

**Affiliations:** 1 College of Mechanical Engineering, Guangdong Ocean University, Zhanjiang 524088, China; gechen@gdou.edu.cn (G.C.); neomailphc@gdou.edu.cn (H.P.); wangxianzhang@gdou.edu.cn (X.W.); 2 Guangdong Provincial Key Laboratory of Intelligent Equipment for South China Sea Marine Ranching, Guangdong Ocean University, Zhanjiang 524088, China; 3 College of Naval Architecture and Shipping, Guangdong Ocean University, Zhanjiang 524088, China

**Keywords:** wettability surfaces, droplet manipulation, contact hysteresis, bioinspired self-propulsion, external field regulation

## Abstract

Firstly, this paper reviews the fundamental theories of solid surface wettability and contact angle hysteresis. Subsequently, it further introduces four typical wettability-engineered surfaces with low hysteresis (superhydrophobic, superamphiphobic, super-slippery, and liquid-like smooth surfaces). Finally, it focuses on the latest research progress in the field of droplet manipulation on open planar surfaces with engineered wettability. To achieve droplet manipulation, the core driving forces primarily stem from natural forces guided by bioinspired gradient surfaces or the regulatory effects of external fields. In terms of bioinspired self-propelled droplet movement, this paper summarizes research inspired by natural organisms such as desert beetles, cacti, self-aligning floating seeds of emergent plants, or water-walking insects, which construct bioinspired special gradient surfaces to induce Laplace pressure differences or wettability gradients on both sides of droplets for droplet manipulation. Moreover, this paper further analyzes the mechanisms, advantages, and limitations of these self-propelled approaches, while summarizing the corresponding driving force sources and their theoretical formulas. For droplet manipulation under external fields, this paper elaborates on various external stimuli including electric fields, thermal fields, optical fields, acoustic fields, and magnetic fields. Among them, electric fields involve actuation mechanisms such as directly applied electrostatic forces and indirectly applied electrocapillary forces; thermal fields influence droplet motion through thermoresponsive wettability gradients and thermocapillary effects; optical fields cover multiple wavelengths including near-infrared, ultraviolet, and visible light; acoustic fields utilize horizontal and vertical acoustic radiation pressure or acoustic wave-induced acoustic streaming for droplet manipulation; the magnetic force acting on droplets may originate from their interior, surface, or external substrates. Based on these different transport principles, this paper comparatively analyzes the unique characteristics of droplet manipulation under the five external fields. Finally, this paper summarizes the current challenges and issues in the research of droplet manipulation on the open planar surfaces and provides an outlook on future development directions in this field.

## 1. Introduction

Droplet manipulation refers to the process of guiding droplets to move along predetermined paths through precisely designed surface physical/chemical properties or applying external forces. As a crucial branch of wettability surface science [1,2], droplet manipulation demonstrates broad application potential in various fields, which includes but is not limited to applications such as SERS biochemical sensing in digital microfluidics systems [3,4,5,6,7], water harvesting [8,9,10,11,12], efficient oil–water separation [13,14,15], microbiological diagnosis in clinical settings [16], single-cell analysis and immunotherapy during treatment [17], and heat transfer scenarios [18,19,20,21,22].

In the exploration of droplet manipulation, biomimetic concepts serve as a guiding light to illuminate the path forward. Researchers draw inspiration from natural phenomena, which mainly include the self-cleaning surface of lotus leaves [23,24], the waterproof and oil-repellent skin of springtails [25,26], the insect-trapping mechanism of pitcher plants [27,28], the water-collecting strategy of cacti [29,30], self-migration behavior of floating seeds via emergent aquatic plants [31,32], and the meniscus-climbing behavior of some insects [33,34]. These unique surface characteristics of organisms have been ingeniously mimicked and applied in the research of passive droplet manipulation [35,36], which enable bioinspired structures to achieve spontaneous droplet propulsion.

Moreover, recognizing the limitations of passive droplet manipulation in certain scenarios, researchers have actively explored novel methods for active droplet manipulation [37,38,39] under external stimuli, such as electric fields, thermal fields, optical fields, acoustic fields, and magnetic fields. **Benefiting from** the application of these external fields, the flexibility, speed, and transport distance of droplets have been significantly enhanced, which enables robust support for technological advancements in related fields.

This paper aims to comprehensively review two core research directions in the field of wettability surfaces: (1) passive droplet manipulation (self-propelled droplet manipulation based on bioinspired gradient surfaces) and (2) active droplet manipulation (external field-assisted droplet manipulation). Thus, this article begins with a detailed introduction to the theory of surface wettability [40,41] and the typical barriers (contact angle hysteresis) [40] that must be overcome for droplet initiation. On this basis, this article further elaborates in detail on four major types of surfaces with low contact angle hysteresis.

Subsequently, this article delves into an in-depth discussion of passive and active droplet manipulation research. It highlights various actuation mechanisms proposed to overcome surface contact angle hysteresis and provides detailed examples to elaborate on the implementation strategies of each transport method, as well as their advantages and limitations in droplet manipulation. Through comparison and analysis, this study further elucidates the commonalities and differences in droplet manipulation across different research pathways. Whether the passive strategies that mimic the surface characteristics of organisms in nature or the active strategies using external fields, each is presented and meticulously analyzed.

Finally, this article reflects on the current challenges in the field of droplet manipulation on wettability surfaces and provides an outlook on future development trends. By summarizing and evaluating existing research, this article aims to present readers with a comprehensive overview of the studies on droplet manipulation, which enable foster a deeper understanding and exploration of the field and provide valuable references and insights for future scientific research and technological applications.

## 2. Wettability and Contact Angle Hysteresis

To quantitatively characterize the wetting behavior of liquids on ideal surfaces, Young proposed Young’s equation in 1805. Subsequently, Cassie and Baxter developed the Cassie–Baxter model based on Young’s equation, which was to further describe the relationship between surface roughness and wettability on **liquid-repellent surfaces**.

### 2.1. Young’s Equation

**Figure 1:**
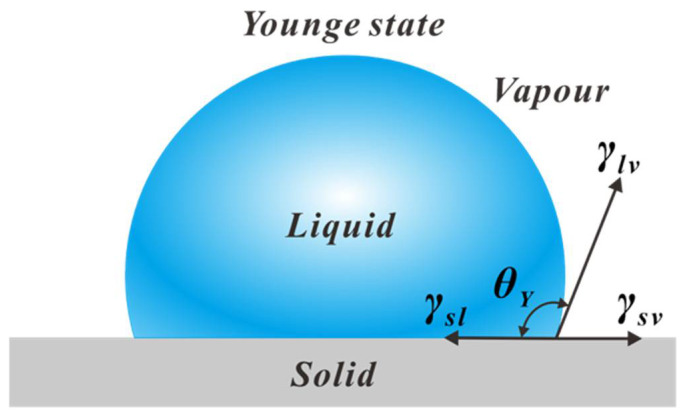
Schematic illustration of Young’s equilibrium state for a droplet on an ideal smooth substrate.

In 1805, Young [40,42,43] proposed that the contact angle could serve as an important indicator for describing the extent to which a liquid wets a solid surface. When a droplet is deposited on an ideal planar surface as shown in Figure 1, The contact angle refers to the angle formed at the three-phase boundary (solid, liquid, gas) where the gas–liquid interface meets the solid–liquid interface. Thus, the contact angle can be analyzed by considering the three interfacial tensions acting on the droplet. When these three forces reach equilibrium, the contact angle $\theta_{Y}$ is defined as

(1) $$\cos\theta_{Y}=\frac{\gamma_{sv}-\gamma_{sl}}{\gamma_{lv}}$$

where $\gamma_{sv},\gamma_{sl}$, and $\gamma_{lv}$ represent the interfacial tensions at the solid–vapor, solid–liquid, and liquid–vapor interfaces, respectively. It should be noted that Young’s equation applies only to ideal surfaces, which are chemically homogeneous, smooth, rigid (not deformable under the normal component of liquid surface tension), and isotropic. Only on such surfaces can a solid surface exhibit a fixed equilibrium contact angle as shown in Young’s equation.

### 2.2. Contact Angle Hysteresis

For an ideally smooth and homogeneous surface, the contact line of a droplet would remain unpinned, allowing for the droplet to move freely at any inclination angle. However, such ideal conditions are rarely achieved due to inevitable chemical and physical heterogeneities. At the atomic scale, the contact line becomes pinned to the substrate, which is commonly known as contact line pinning [44,45].

**Figure 2:**
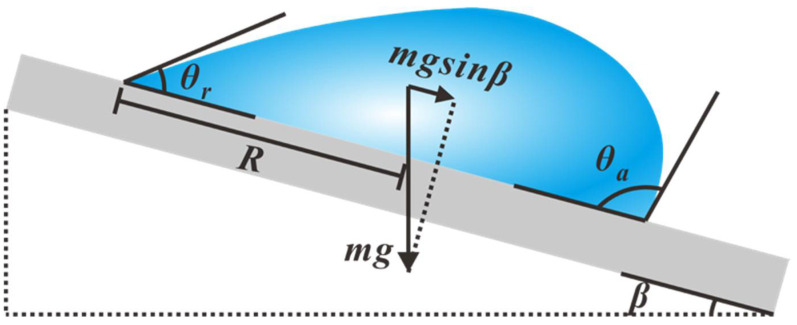
Schematic showing contact angle hysteresis: a droplet on a slope starts moving when gravity-induced deformation surpasses the hysteresis-permitted contact angle range.

As shown in Figure 2, a droplet with mass m and base radius R is placed on an inclined surface. Due to the presence of contact line pinning, there exists a critical tilt angle β at which the droplet just begins to slide relative to the substrate. During droplet motion, contact line friction induces the formation of two distinct contact angles: the larger advancing contact angle (*θ_a_*) at the front and the smaller receding contact angle (*θ_r_*) at the rear, which is defined as the phenomenon of contact angle hysteresis (CAH) [46].

Contact angle hysteresis [47] represents the primary resistance to droplet motion on solid surfaces. Based on force balance analysis, the lateral adhesion force ($F_{laf}$) opposing droplet motion can be expressed as

(2) $$F_{laf}=mg\sin\beta =kR\gamma_{la}\left(\cos\theta_{r}-\cos\theta_{a}\right)$$

Herein, *k* is a dimensionless geometric factor (determined by the specific morphology of the solid–liquid–gas three-phase contact line), with values ranging between 1 and π; *g* is the gravitational acceleration constant; $\gamma_{la}$ denotes the liquid–air interfacial tension.

### 2.3. Liquid-Repellent Surfaces with Low Contact Angle Hysteresis

#### 2.3.1. Superhydrophobic Surface (Non-Wetting Cassie State)

**Figure 3:**
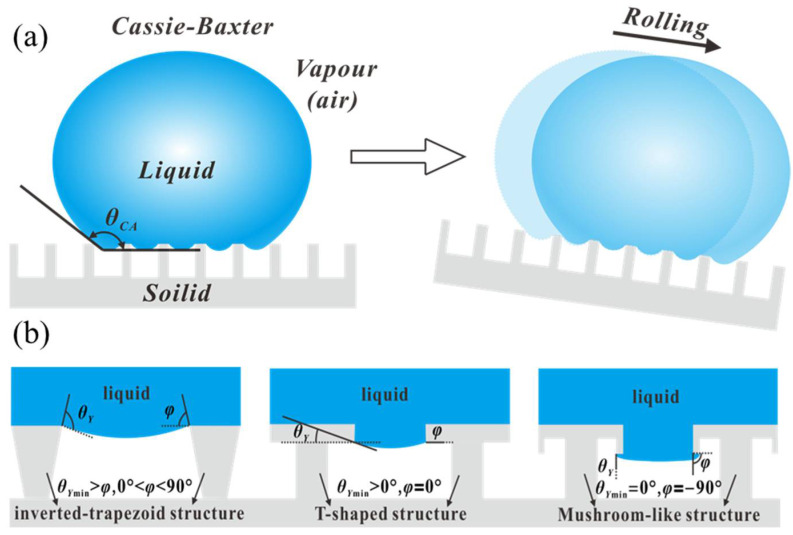
(**a**) Schematic illustration of a water droplet in the non-wetting Cassie state on a structured superhydrophobic surface, showing low contact angle hysteresis. (**b**) The schematic from left to right illustrates the conditions for the minimum intrinsic contact angle ($\theta_{Ymin}$) required to maintain the metastable Cassie state on the three typical reentrant structures: inverted trapezoid ($\theta_{Ymin}>\varphi$, $0^{{}^{\circ}}>\varphi <90^{{}^{\circ}}$), T-shape ($\theta_{Ymin}>0^{{}^{\circ}}$, $\varphi =0^{{}^{\circ}}$), and mushroom-like ($\theta_{Ymin}=0^{{}^{\circ}}$, $\varphi ={-90}^{{}^{\circ}}$) configurations.

To reduce the lateral adhesion force caused by contact angle hysteresis. Such people as Zorba, V., ratakis, E., as well as Li, P., Wang, L. and Zhao, F. et al. drew inspiration from lotus leaves [23,24], which was that a spherical droplet can rest and roll away easily on a rough surface with sufficiently low surface energy. For the reason, it is that the liquid cannot penetrate the gaps between microstructures due to capillary effects, which results in trapped air pockets within these gaps. As described by the Cassie–Baxter model [48] Figure 3a, the droplet can be suspended atop the microstructures. Consequently, the contact angle $\theta_{CA}$ can be defined as the weighted average of the contact angles on the solid and air, expressed by the following equation:

(3) $$\cos\theta_{CA}=f_{sl}\cos\theta_{Y}+f_{lv}\cos\theta_{air}\;$$

Here, $f_{sl}$ and $f_{lv}$ represent the area fractions of the liquid–solid and liquid–vapor interfaces under the droplet, respectively, ($f_{sl}{+f}_{lv}=1$). $\theta_{Y}$ is the Young’s contact angle on a smooth solid surface, and $\theta_{air}$ is the contact angle of air within the microstructures, which can be approximated as 180°. Accordingly, the Cassie–Baxter equation can be rewritten as

(4) $$\cos\theta_{CA}=\left.f_{\mathrm{sl}}(\cos\theta_{Y}\right.+1)-1$$

Based on this equation, when $\theta_{Y}$ > 90° (the original surface is hydrophobic), $\theta_{CA}$ always remains greater than $\theta_{Y}$. As the liquid–solid contact area fraction ($f_{sl}$) decreases, the surface can eventually exhibit superhydrophobicity ($\theta_{CA}\geq 149{}^{\circ}$). The significant increase in contact angle greatly reduces the contact area between the droplet and the substrate, while the presence of air pockets further minimizes adhesion. As a result, contact angle hysteresis on superhydrophobic surfaces is substantially reduced to less than 10°.

#### 2.3.2. Superomniphobic Surface (Metastable Cassie State)

Although many commercial hydrophobic materials combined with rough surfaces can easily achieve superhydrophobicity, most low-surface-tension liquids still exhibit wetting behavior on existing hydrophobic materials. Clearly, designing surfaces capable of repelling a wide range of low-surface-tension liquids poses a significant challenge. Inspired by the super-oleophobic properties of springtail skin, researchers have discovered that when the minimum geometric angle (φ) of a reentrant structure is smaller than the intrinsic contact angle ($\theta_{Y}$) of the liquid on the surface ($\theta_{Y}>\varphi$) [49], the free liquid meniscus between structures can generate upward capillary forces through its own curvature, which enable prevent water and oil from penetrating into the rough micro- or nanostructures. Here, the minimum geometric angle (φ) is defined as the angle between the tangent of the structural profile and the horizontal axis, which typically occurs at the edges of the structure.

As illustrated in Figure 3b, three representative reentrant structures with concave geometries are presented [50]. Evidently, as the structure evolves from an inverted trapezoid to a T-shape and finally to a mushroom-like configuration, the progressive decrease of the minimum geometric angle (φ) is to mean that the minimum intrinsic contact angle (θ*_Ymin_*) required to sustain the droplet in a metastable Cassie state will likewise drop rapidly. For example, the required $\theta_{Ymin}$ for inverted trapezoidal structures is $\theta_{Ymin}>\varphi$, where $0^{{}^{\circ}}>\varphi <90^{{}^{\circ}}$, and the required $\theta_{Ymin}$ for T-shape structures is $\theta_{Ymin}>0^{{}^{\circ}}$, where $\varphi =0^{{}^{\circ}}$. Particularly, the mushroom-like structure ($\theta_{Ymin}=0^{{}^{\circ}}$, $\varphi ={-90}^{{}^{\circ}}$) can theoretically repel any liquid with a contact angle hysteresis of less than $10^{{}^{\circ}}$. However, the fabrication complexity also increases accordingly.

Furthermore, modifying the structure with hydrophobic materials can enhance the intrinsic contact angle [28], thereby increasing the maximum curvature of the free liquid meniscus and strengthening the capillary forces that repel liquids. To meet the demands of different applications, combining hydrophobic materials with rationally designed geometries ensures that the liquid remains suspended on the structure through upward capillary forces (metastable Cassie state), which results in super-repellency with high contact angles and low hysteresis for the test liquid.

#### 2.3.3. Omniphobic Super-Slippery Surface

**Figure 4:**
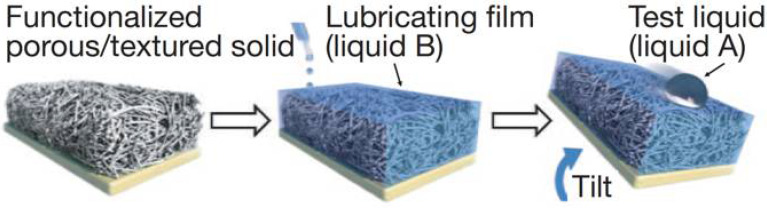
Schematic illustration of the fabrication of a super-slippery surface by infusing a chemically inert liquid into a structured substrate with low surface energy to form a physically smooth and chemically homogeneous lubricating film. Reproduced with permission from Ref. [28]. Copyright 2011 Nature.

Inspired by the pitcher plant, the Aizenberg’s research group [28] pioneered the design of a novel, low-hysteresis omniphobic surface (Figure 4), naming it the “Slippery Liquid-Infused Porous Surface” (SLIPS). To rationally design SLIPS, three key criteria must be met: 1. The lubricant must wet the surface and infiltrate the voids of the structured substrate. 2. The substrate should be preferentially wetted by the lubricant rather than the test fluid. 3. The lubricant and working fluid must be immiscible.

To fulfill these requirements, low-surface-energy structural materials (e.g., porous Teflon membranes) with high affinity for the lubricant are used to lock the lubricant in place. When the lubricant is coated onto the porous substrate, capillary forces draw it into the matrix to form a smooth and stable lubricating layer. For the lubricating layer, typical low-surface-tension perfluorinated liquids (3M Fluorinert FC-70 or DuPont Krytox oils) [51,52] are commonly employed. These lubricants are non-volatile and immiscible with both aqueous and hydrocarbon phases, which makes SLIPS adaptable to a wide range of polar and nonpolar liquids including water, acids, bases, alkanes, alcohols, and ketones. Since the resulting SLIPS exhibits a molecularly smooth and homogeneous surface with a roughness of ~1 nm, it demonstrates an extremely low contact angle hysteresis (Δθ ≤ 2.5°).

#### 2.3.4. Omniphobic Liquid-Like Smooth Surface

To address the issues of lubricant loss and medium contamination caused by droplet transport on super-slippery surfaces, an alternative strategy involves covalently grafting flexible polymer brushes (e.g., polydimethylsiloxane (PDMS) and perfluoropolyether (PFPE)) onto smooth surfaces for liquid repellency [53].

**Figure 5:**
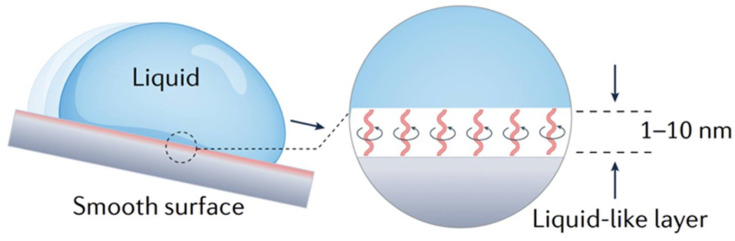
Schematic illustration of the omniphobic liquid-like smooth surface with grafted polymer brush. Reproduced with permission from Ref. [53]. Copyright 2023 Nature Reviews Chemistry.

As is shown in Figure 5, one end of the polymer chains is firstly covalently anchored to the substrate surface, which ensures that these molecular structures remain fixed and cannot be dissolved or displaced by contacting liquids. Meanwhile, the free ends of the polymer chains exhibit high mobility at room temperature, which enable the formation of a liquid-like lubricating layer. This layer can adapt to liquids of varying surface tensions and demonstrates ultra-low contact angle hysteresis (i.e., the difference between advancing and receding contact angles, which characterizes the frictional resistance of the surface against droplets).

## 3. Bioinspired Passive Manipulation on Open Planar Surfaces with Engineered Wettability

Based on fundamental physical principles, the droplet can be moved once the driving force overcomes contact angle hysteresis on the droplet. In nature, natural organisms directionally transport target liquids by rationally manipulating surface energy of droplet through gradient surfaces for survival [32,33,54,55,56,57]. Learning from and surpassing nature, droplet manipulation has now evolved into two main categories: passive and active manipulation. Among them, passive manipulation inspired by nature requires no external stimulation, which instead relies solely on droplet interactions with the surface to achieve spontaneous motion. Currently, bioinspired passive manipulation mainly includes the following three types:

### 3.1. Wettability-Gradient-Induced Manipulation

**Figure 6:**
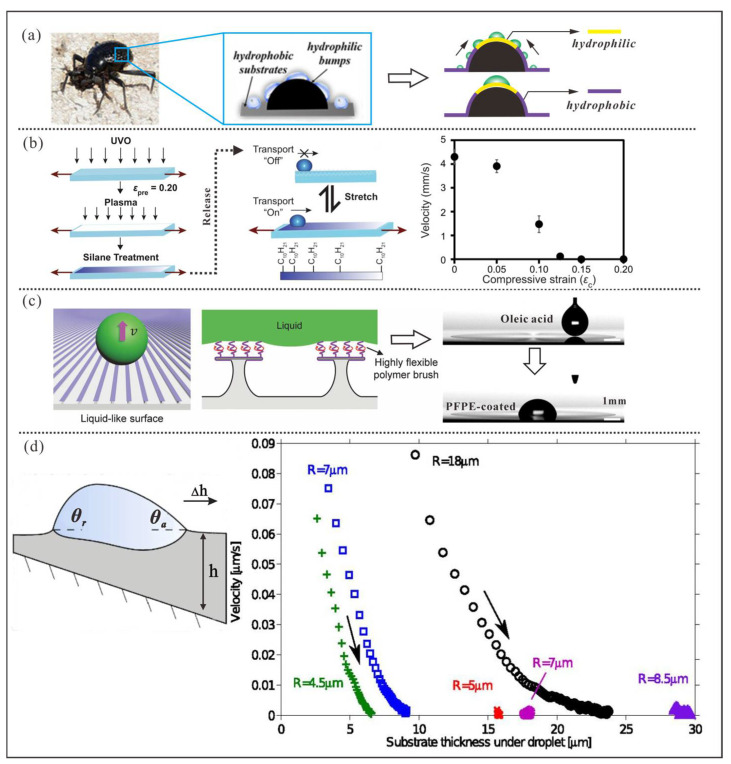
Droplet manipulation based on wettability gradients. (**a**) Desert beetles rely on hydrophobic–hydrophilic patterns on their dorsal surfaces to achieve water-harvesting capabilities. Reproduced with permission from Ref. [8]. Copyright 2019 PNAS. (**b**) Droplet manipulation using a radial gradient of T-shaped microstrip arrays modified with liquid-like coatings. Reproduced with permission from Ref. [65]. Copyright 2021 Nature Communications. (**c**) Droplet manipulation via mechanically tunable surface chemical composition gradients. Reproduced with permission from Ref. [70]. Copyright 2019 WILEY-VCH. (**d**) Droplet transport driven by stiffness gradients on soft substrates with thickness variations. Reproduced with permission from Ref. [74]. Copyright 2013 PNAS.

To survive in extremely arid environments, the desert beetle’s back [8,58,59] is covered with numerous protrusions (Figure 6a) [8]. The top of each protrusion exhibits hydrophilicity, while the sloping sides are coated with a hydrophobic substance. When the beetle is exposed to humid air, the asymmetric deformation of droplets on the wettability gradient surface ($\theta_{ad}<\;\theta_{rd}$) induces unequal internal pressure to force the droplet to migrate toward regions with higher wettability, which facilitates the nucleation and condensation of water in hydrophilic areas. Inspired by such biological mechanisms, three artificial strategies based on regulated chemical functional groups [60,61,62,63,64,65,66,67,68] or microstructures [69,70,71,72,73] on surfaces, and the thickness distribution of soft substrates [74,75,76,77] have been developed and applied. On such solid surfaces, the wettability gradient-induced driving force, $F_{WD}$, can be expressed as follows [60,64]:

(5) $$F_{WD}=\pi {R_{b}}^{2}\gamma k\sin\theta_{d}$$

where cosθd=12(cosθa+cosθr). Here, $\theta_{d}$ represents the dynamic contact angle of the droplet; $\theta_{a}$ and $\theta_{r}$ denote denote the advancing and receding contact angles at the front and rear ends of the droplet, respectively; $k=-d\theta_{d}/dx$ is the spatial gradient of wettability (usually a preset constant); γ is the liquid’s surface tension; and $R_{b}$ is the base radius of the droplet.

Initially, when $F_{WD}$ is sufficiently large the droplet can overcome the resistance caused by contact angle hysteresis and begin moving. However, as $\theta_{d}$ gradually decreases and approaches, and the resistance increases with the expanding contact area, the droplet’s effective transport distance becomes limited due to forced termination of motion. Although increasing k can enhance $F_{WD}$ and improve droplet velocity and improve droplet velocity, it simultaneously further restricts the transport distance.

#### 3.1.1. Chemical Gradient-Based Droplet Manipulation

As shown in Figure 6b, Ali et al. [65] first treated a 20% pre-stretched PDMS film with UVO (an oxidation method that generates a micron-scale silica surface layer) to induce controlled micro-wrinkles upon strain release. Following UVO treatment, the film was briefly exposed to oxygen plasma to enhance surface silanol concentration for facilitating subsequent fluor silane vapor deposition. Finally, a chemically gradient surface was built on the substrate via template-guided chemical vapor diffusion. As a result, the chemical gradient—correlated with the spacing and amplitude of micro-wrinkles—could be dynamically tuned by mechanical stretching. As a result, the gradient could be flexibly adjusted through mechanical stretching, which enables on-demand droplet transport and positioning. However, the average speed remained at a few millimeters per second, and the effective transport distance was limited to around 10 mm.

#### 3.1.2. Microstructure Gradient-Based Droplet Manipulation

As shown in Figure 6c, Huang et al. [70] fabricated a radially inward-distributed T-shaped microstrip array on a silicon wafer using photolithography and dry etching techniques. This structure was designed to create a wettability gradient induced by a continuous reduction in the liquid–gas contact fraction along the radial inward direction. By further grafting liquid-like polymer brushes onto the microstructured surface, they achieved self-transport of droplets with surface tensions ranging from 19.8 to 65.3 mN·m^−1^. However, in performance tests, the maximum transport speed for oleic acid droplets was only 27.5 mm/s, and the transport distance was limited to 2.3 mm.

#### 3.1.3. Thickness Gradient-Based Droplet Manipulation

As shown in Figure 6d, Style, R. W. et al. placed droplets on a silicone gel layer with a continuous stiffness gradient [74], which was inspired further by the durotactic behavior of biological cells [78]. Since the material’s stiffness varies with thickness and the droplet’s internal pressure at the interface competes with the substrate’s stiffness, the droplet ultimately spontaneously achieves equilibrium by sinking deeper into regions of lower stiffness. Consequently, the substrate’s stiffness gradient translates into a contact angle gradient of the droplet, which drives the droplet toward softer regions. However, the droplet transport speed on such surfaces never exceeded 100 nm/s.

### 3.2. Surface Shape Gradient-Induced Manipulation

**Figure 7:**
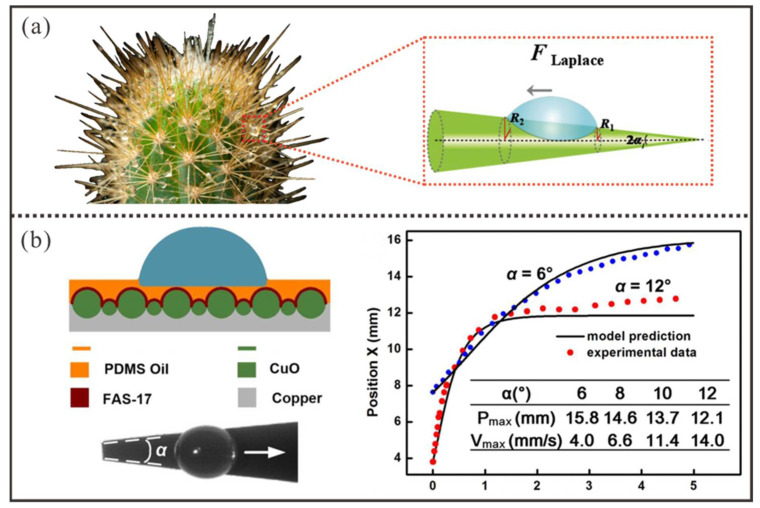
Droplet manipulation based on shape gradient: (**a**) Cactus utilizes conical spines for directional water droplet collection and transport. Reproduced with permission from Ref. [84]. Copyright 2013 WILEY-VCH. (**b**) Droplet control using V-shaped super-slippery surfaces. Reproduced with permission from Ref. [88]. Copyright 2017 American Chemical Society.

In addition to the aforementioned actuation mechanisms based on surface wettability gradients, special bioinspired surfaces with shape gradients [79,80,81,82,83,84,85,86,87,88] are also widely utilized to achieve droplet manipulation by inducing curvature gradients on droplets. As illustrated in Figure 7a [84], cacti in deserts employ their conical spines to directionally collect and transport water droplets. On such surfaces, the droplet undergoes asymmetric deformation due to the surface shape gradient, which generates a curvature gradient between two ends of the droplet to produce a Laplace pressure-driven force ($F_{Laplace}$) [80,83] expressed as

(6) FLaplace≈γ·sin2α1·1R1R2·Ω

Here, Ω represents the droplet volume, $\alpha_{1}$ represents the half-apex angle of the conical substrate, and $R_{1}$ and $R_{2}$ denote the local radii of curvature at both ends of the receding and advancing contact lines, respectively. When Ω is sufficiently large, $F_{Laplace}$ can overcome resistance to cause the droplet to move along the wedge toward the widening direction. However, $R_{1}$ and $R_{2}$ gradually increase as the droplet advances, which leads to a rapid decline in $F_{LD}$. Meanwhile, the resistance rises due to the expanding contact area. Eventually, when the driving force falls below resistance, the droplet is forced to halt with a limited transport distance.

As shown in Figure 7b [88], to achieve directional water droplet transport, Luo et al. designed a wedge-shaped substrate with a super-slippery surface. Herein, Luo et al. utilized alkaline materials (NaOH and (NH_4_)_2_S_2_O_8_) to chemically etch a copper foil to form the CuO microstructures, which could then be further modified with a fluorosilane (FAS) to reduce the surface energy. Subsequently, PDMS oil was infused into the modified CuO microstructures to create a desired super-slippery surface (SLIPS). When droplets are deposited at the narrow end of the wedge-shaped surface, they are driven from the narrow region to the wider region by Laplace pressure difference. All droplets achieved directional movement on the wedge surface within the wedge angle range of α = 6–12°. The experiment shows that when the droplet was deposited on the horizontal wedge-shaped surface with an angle (α) ranges from 6° to 12°, the maximum speed increases from 4.0 mm/s to 14.0 mm/s whereas the corresponding transport distance decreases from 15.8 mm to 12.1 mm.

### 3.3. Asymmetric Meniscus Curvature-Induced Manipulation

**Figure 8:**
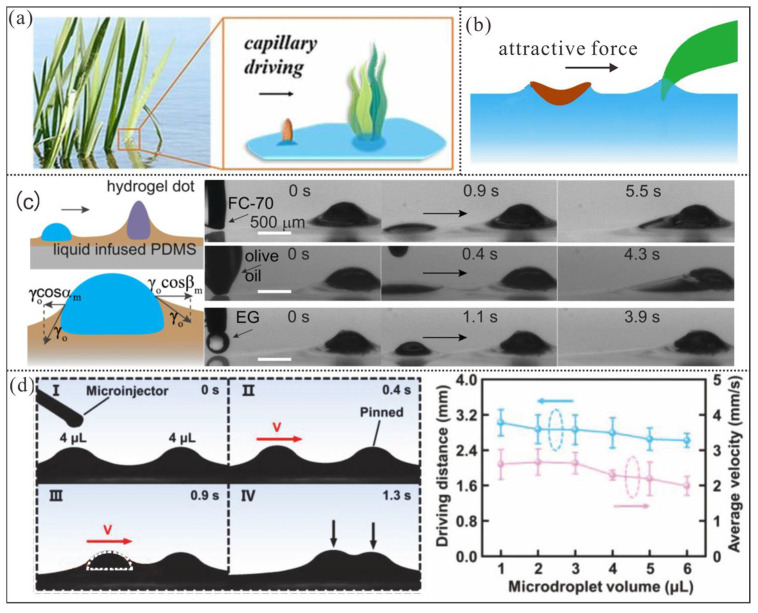
Droplet manipulation based on asymmetric meniscus-induced capillary forces: (**a**) Seed dispersal: Seeds achieve dispersal through the net capillary force induced by the meniscus between themselves and emergent aquatic plants. Reproduced with permission from Ref. [8]. Copyright 2019 PNAS. (**b**) Insect landing: Certain insects deform the surrounding free water surface to alter the meniscus, enabling “landing” via capillary attraction between their bodies and the meniscus near solid surfaces (“land”). Reproduced with permission from Ref. [89]. Copyright 2022 Nature Chemistry. (**c**) Droplet manipulation: Mimicking the seed-capturing behavior of emergent aquatic plants, droplets are manipulated on a super-slippery surface with localized hydrogel protrusions. Reproduced with permission from Ref. [90]. Copyright 2019 PNAS. (**d**) Rapid droplet collection: Droplets on the surrounding super-slippery surface are rapidly collected using localized fixed lubricant droplets. Reproduced with permission from Ref. [91]. Copyright 2022 WILEY-VCH.

Additionally, Peruzzo, P., Defina, A. et al. have discovered that the floating plant seeds and certain insects (such as water striders and beetle larvae) can utilize surface tension of water to achieve self-propelled movement [8,32] (Figure 8a) and meniscus-climbing [33,89] (Figure 8b), which enables them to transfer from the water to solid objects for accomplishing dispersal and landing. The underlying actuation mechanism is similar to the well-known Cheerios effect [57], where the interaction between the floating object and nearby emergent protrusions creates an imbalance in the curvature of the meniscus on either end of the object, which results in a net capillary driving force.

Inspired by the aforementioned phenomena, Jiang et al. [90] fabricated a super-slippery surface with hydrogel protrusions on a PDMS substrate using printing and swelling-filling techniques (Figure 8c). Thanks to the capillary driving force, they realized the droplet transport and conducted an in-depth study on the relevant dynamic characteristics.

The experimental results indicate that once the menisci induced by the droplet and the fixed protrusion on the lubricating liquid overlapped, the curvature of the menisci on both sides of the droplet became asymmetric. This asymmetry led to a difference in the liquid contact angles ($\alpha_{m}$ and $\beta_{m}$) on either side of the droplet, which resulted in a capillary attraction effect from the fixed protrusion on the droplet. Therefore, the maximum effective **transport** distance is the capillary length of the lubricant, λo=γo/ρo·g12, and the capillary driving force is $F_{CD}=\gamma_{o}R\left(\cos\alpha_{m}-\cos\beta_{m}\right)$. Meanwhile, recent studies have shown that the total dissipative force acting on a moving droplet on a super-slippery surface is FTd=2Rγlo13ηoV23. Thus, the net force on the droplet is $F=F_{CD}-F_{Td}$. Here, $\gamma_{o}$ and $\rho_{o}$ denote the surface tension and density of the lubricating oil, respectively; g is gravitational acceleration; $\alpha_{m}$ and $\beta_{m}$ represent the inclination angles of the lubricating oil menisci at both ends of the droplet in the horizontal direction; $\gamma_{lo}$ is the interfacial tension between the lubricating oil and the droplet; and $\eta_{o}$ is the viscosity of the lubricating oil. From the net force equation, it is evident that the droplet’s motion speed is closely related to the viscosity and surface tension of the lubricating oil.

However, the need to accommodate a variety of liquids often requires the use of fluorinated lubricating oils with extremely low surface tension, which limits the driving force. Additionally, to reduce the motion resistance of droplets, the viscosity of the chosen lubricating oil must also be relatively low. In the example, Jiang et al. used 10 cSt silicone oil as the lubricating fluid. Nevertheless, the average controllable speed of the droplets was generally below 1 mm/s. Moreover, Wang et al. [91]. recently trapped droplets on superhydrophilic spots of a substrate and infused 10 cSt silicone oil into the adjacent superhydrophobic areas to create a super-slippery surface (Figure 8d). As a result, droplets on the super-slippery surface could be transported to the trapped droplet via capillary driving force induced by the wetting ridge. Experimental results showed that the average manipulation speed of the droplets remained below 4 mm/s. However, the volatilization loss of low-viscosity lubricating oil can easily lead to the failure of the super-slippery surface properties. Therefore, the contradiction between actuation speed and lubricant viscosity is another major challenge that needs to be overcome in such methods.

## 4. Active Manipulation on Planar Surfaces with Engineered Wettability

The passive manipulation of droplets is a clever and power-free pumping method that takes advantage of the inherent surface tension of droplets, featuring simplicity, ease of use, and low cost. However, due to the lack of reconfigurability and certain limitations (controllable direction, spatiotemporal precision, transmission distance, and speed), it is only suitable for applications with simple steps and low precision requirements. Therefore, active strategies utilizing various external physical fields have been developed to enhance manipulation performance and better meet application demands. Especially in digital microfluidic systems, droplet-based manipulation and classification such as droplet sorting [92] and droplet splitting [93] are fundamental issues. Thus, according to the type of external stimulus, current active methods can be classified into electrical, thermal, optical, acoustic, and magnetic manipulation.

### 4.1. Electrical Manipulation

According to whether the driving force is generated at the contact interface, it is categorized into electrowetting-based [94,95,96,97,98,99,100,101,102,103,104] and electrostatic-based [105,106,107,108,109] manipulation.

#### 4.1.1. Electrowetting-Based Manipulation

**Figure 9:**
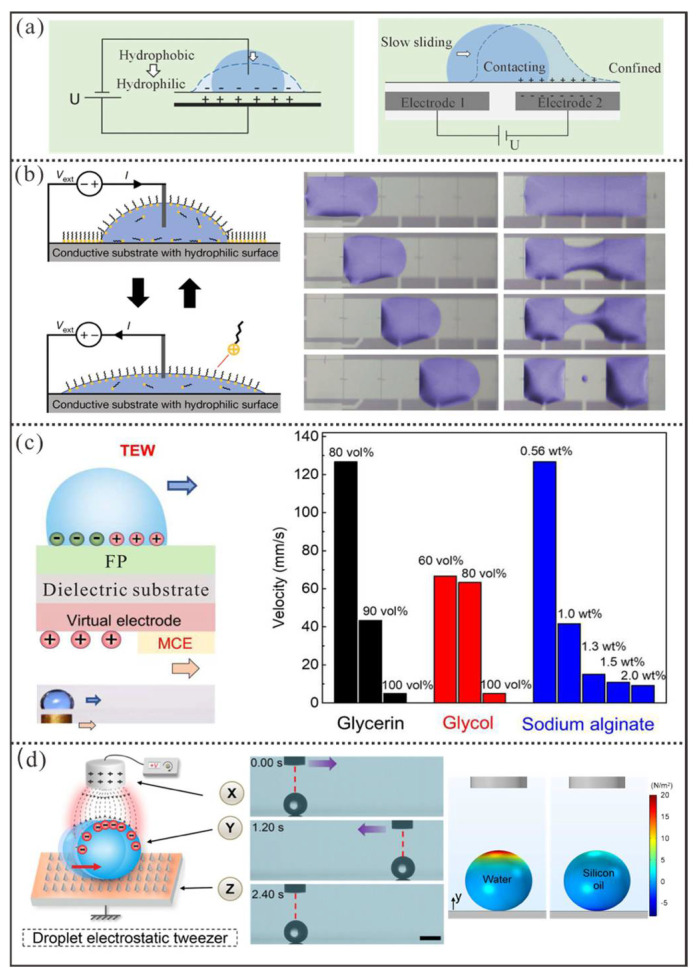
Electrical manipulation of droplets: (**a**) Droplet manipulation based on conventional electrowetting. Reproduced with permission from Ref. [94]. Copyright 2020 CHEMICAL JOURNAL OF CHINESE UNIVERSITIES. (**b**) Droplet manipulation mediated by ionic surfactants on a conductive hydrophilic substrate via electrical dewetting. Reproduced with permission from Ref. [101]. Copyright 2019 NATURE. (**c**) Droplet manipulation based on triboelectric wetting. Reproduced with permission from ref. [102]. Copyright 2022 SCIENCE ADVANCES. (**d**) Droplet manipulation using electrostatic tweezers on a superamphiphobic metal substrate. Reproduced with permission from Ref. [108]. Copyright 2022 PNAS.

As illustrated in Figure 9a [94], the principle of electrowetting-on-dielectric (EWOD) involves modulating droplet wettability by adjusting the voltage applied between the droplet and an electrode beneath an insulating layer. In the application of droplet manipulation, the surface of the insulating layer will be further coated with a hydrophobic layer to minimize droplet motion resistance. Thus, switching a series of adjacent microelectrodes on and off in a specific order allows for on-demand generation of a directional and tunable wettability gradient, which enable programmable droplet control. According to the Lippmann–Young equation, the contact angle (θ) of a droplet under electrowetting can be expressed as [97]

(7) $$\cos\theta =\cos\theta_{Y}+\frac{1}{2}\frac{\varepsilon_{0}\varepsilon_{d}}{d\gamma }V^{2}$$

Here, $\theta_{Y}$ is the Young’s contact angle without applied voltage; γ denotes the surface tension of the droplet; $\varepsilon_{0}$ and $\varepsilon_{d}$ are the permittivities of vacuum and the dielectric layer, respectively; d is the thickness of the insulating layer; and V is the applied voltage. Clearly, the actual electrowetting performance largely depends on the physical properties of the droplet itself. Therefore, high actuation voltages (above 90 volts) [98] are often required for many liquids with low conductivity and permittivity. However, high voltage can easily lead to dielectric breakdown and unwanted adsorption of biomolecules onto the substrate surface [99], which compromises the fidelity of subsequent analytical tests. Additionally, it may cause satellite droplet ejection [100].

To avoid various challenges associated with high-voltage electrowetting, Li et al. [101] recently proposed novel ion-surfactant-mediated electrowetting–dewetting technology (Figure 9b). Firstly, an aqueous droplet containing ionic surfactant molecules is spread on a hydrophilic, smooth, and conductive substrate. When a direct current is applied across the droplet in in positive or negative direction, hydrophobic ionic surfactant molecules either deposit onto or retreat from the substrate surface under the electric field, enabling dynamic modulation of the droplet’s wetting and dewetting behavior for directional transport and splitting. Since this system requires a low driving voltage (within 5V) and eliminates the need for a dielectric layer or hydrophobic coating, it can simplify the device structure and reduce costs to some extent. However, the introduction of ionic surfactants and the passage of current in the droplet pose new challenges for future applications.

Alternatively, Xu et al. [102] developed a novel droplet manipulation method based on triboelectric wetting (Figure 9c). In this approach, researchers utilized the N-(2-aminoethyl)-3-aminopropyltrimethoxysilane (APTS) coating on an insulating substrate (dielectric layer) as a virtual electrode. Upon contact between the virtual electrode and the metal copper electrode (MCE), electron transfer occurs between them to rapidly increase the surface charge density in the contact region of the virtual electrode, which in turn leads to a reduction in the contact angle of the droplet on the hydrophobic coating marked as FP. Thus, the continuous movement of metal copper electrode causes the virtual electrodes to function like sequentially toggled microelectrodes in conventional electrowetting, which ultimately enable free manipulation of the droplet. Since the system no longer requires complex embedded electrode designs, the related equipment has been significantly simplified. However, this method demands that the substrate be sufficiently thin and is highly sensitive to the physical properties of the droplets themselves, thus posing many constraints. For example, a glass substrate with a thickness of only 200 μm was used in the experiments, and the manipulation speed of droplets containing 80% glycerol could exceed 120 mm/s, whereas droplets with 100% glycerol had a manipulation speed of only about 5 mm/s.

#### 4.1.2. Electrostatic-Based Manipulation

In contrast to the previous electrowetting-driven methods that relies on sufficient contact area for gradient-based actuation, electrostatic actuation of droplets in the Cassie state offers a new approach for electric manipulation. Compared to sliding droplets, spherical droplets in the Cassie state exhibit significantly higher mobility due to the rolling mode. Thus, Jin et al. [108] developed a droplet manipulation system based on electrostatic tweezers (Figure 9d). This system consists of an electrode tip connected to a power supply, the droplet, and a grounded conductive substrate with superamphiphobic coating. When an external voltage is applied to the electrode tip, the electrode can manipulate the droplet like tweezers through the electrostatic coulomb force between them. By moving the tweezers to guide the droplet (guiding mode), the droplet can be transported to any desired location, which achieved a maximum average transport speed of ∼81.6 mm/s for water droplets. However, for silicone oil droplets with a dielectric constant of only about 2.7, whose interaction with the electrostatic tweezer is too weak to be actuated.

### 4.2. Thermal Manipulation

Generally, temperature fields can achieve kinetic energy conversion by altering droplet surface tension or wettability characteristics. Based on different response mechanisms, current approaches primarily fall into two categories, Marangoni flow-driven manipulation [21,110,111,112,113] and wettability gradient-driven manipulation [22,114,115,116,117], which are induced by thermal gradient.

#### 4.2.1. Marangoni Flow-Driven Manipulation

**Figure 10:**
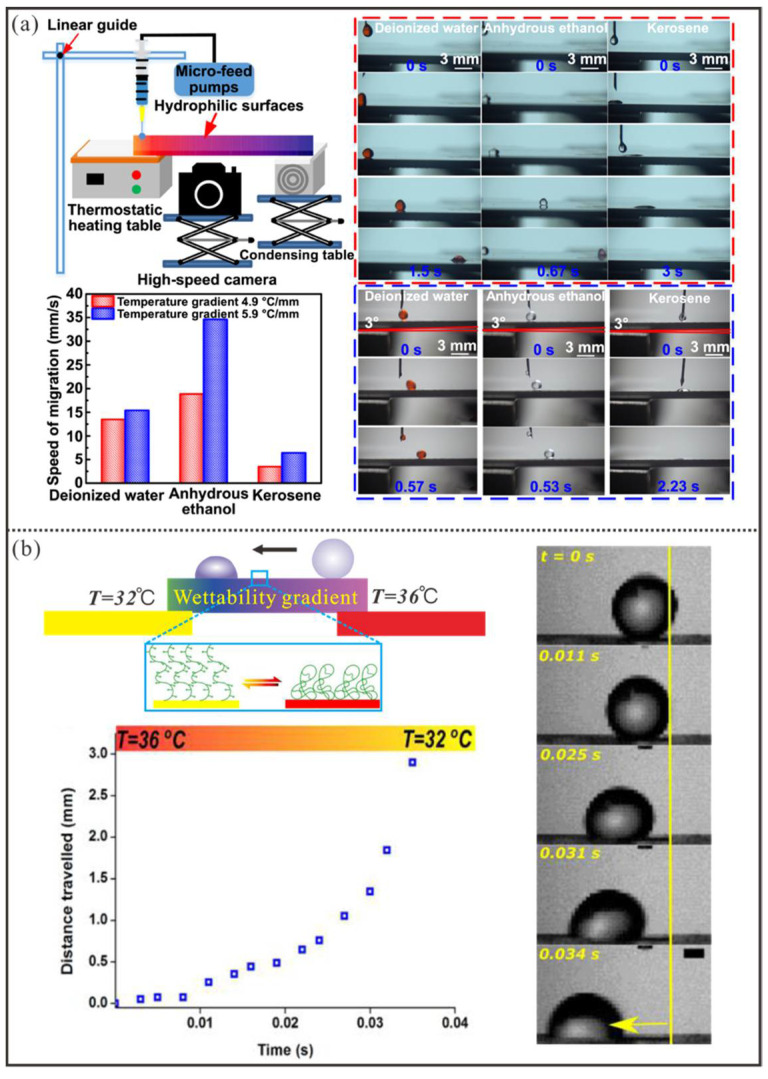
Thermal manipulation of droplets: (**a**) Droplet manipulation driven by thermal-gradient-induced Marangoni flow on a hydrophilic substrate. Reproduced with permission from Ref. [21]. Copyright 2022 IOP Publishing Ltd. (**b**) Droplet manipulation driven by thermal-gradient-induced wettability gradient on a thermoresponsive molecular-modified substrate. Reproduced with permission from Ref. [22]. Copyright 2017 American Chemical Society.

When a droplet resides on a substrate with a linear thermal gradient (∇T), the ∇T generates a spatially distributed surface tension gradient (dγ/dx) along the droplet’s base. The surface tension gradient further triggers Marangoni flow inside the droplet, which produces viscous stress ($F_{M}$) at the droplet’s base for propulsion. As reported [112,113], $F_{M}\approx \pi R^{2}\cdot \frac{d\gamma }{dT}\cdot \frac{dT}{dx}$, where R represents the radii of the droplet base, x denotes the direction of motion, dγ/dT represents the temperature-dependent change in liquid–gas interfacial tension, and dT/dx is the thermal gradient along x. Since for most liquids (dγ/dT<0), the driving force directs toward the cooler side. Recently, Shu et al. [21] developed a platform for droplet transport induced by a temperature gradient on a planar hydrophilic aluminum plate (Figure 10a). In this setup, the two sides of the aluminum plate were in contact with a heating table and a condensing table, respectively. Additionally, the tilt angle of the hydrophilic aluminum plate could be adjusted using a lifting device of the condensing table. Thanks to the vapor layer formed by droplets at high temperatures, the movement friction of droplets was significantly reduced. When the temperatures of the two sides of the aluminum plate were set to 300 °C and 5 °C in the experiment, the controllable speeds of deionized water, anhydrous ethanol, and kerosene were approximately 15 mm/s, 35 mm/s, and 6 mm/s, respectively. Moreover, anti-gravity droplet transport was achieved with a maximum tilt angle of 3°. However, the actuation method relying solely on Marangoni stress faces the issue of an excessively high upper temperature limit, which can easily lead to significant loss of liquid. Moreover, reducing the temperature gradient can lower the upper temperature limit, but the controllable speed of the droplet experiences a substantial decline.

#### 4.2.2. Wettability Gradient-Driven Manipulation

To overcome the challenges existing in Marangoni flow-driven manipulation, Banuprasad et al. [22] grafted a thermosensitive material (Poly(N-isopropylacrylamide)) exhibiting temperature-dependent hydrophobicity onto a textured substrate surface (Figure 10b). When the two ends of the substrate were subjected to temperatures of 32 °C and 36 °C, respectively, the droplet on the functionalized surface was actuated by the predominant wettability gradient to achieve a transport distance of 2.9 mm at an average speed of 8 cm/s. Obviously, the substrate with thermoresponsive materials can produce a temperature-responsive surface wettability gradient for droplet manipulation at lower temperatures, but the droplet can only move along a simple linear trajectory and is confined to a distance of a few millimeters.

### 4.3. Optical Manipulation

The widespread application of light in droplet manipulation has attracted considerable interest due to the capability of non-contact stimulation and the ease of regulating power density, position, and wavelength. Generally, light incident on substrate surfaces cannot directly provide driving force for droplet manipulation. However, the absorbed optical energy can be converted into photothermal, photochemical, or photoelectric energy to achieve the droplet manipulation. According to current excitation light sources, the actuation methods mainly include ultraviolet/blue light-based [117,118,119,120,121,122,123,124], near-infrared light-based [118,125,126,127,128,129,130], and visible light-based [131,132,133] manipulation.

#### 4.3.1. Ultraviolet/Blue Light-Based Manipulation

UV/blue light has demonstrated exceptional potential for droplet manipulation due to the capability to photoactivate molecular responses that alter surface energy. Under asymmetric optical irradiation, droplets undergo directional movement driven by wettability gradient forces. Among photosensitive materials, titanium dioxide (TiO_2_) [117,118,119,120] and azobenzene [121,122,123,124] represent the most prominent candidates. When exposed to UV light, TiO_2_ undergoes a transition from superhydrophobicity to superhydrophilicity. However, its practical application is limited by an excessively long response time (>1 h) and the requirement of specific environmental conditions (e.g., high-temperature treatment or prolonged dark storage) to restore the original wettability state, which hinders continuous operation [118,120].

**Figure 11:**
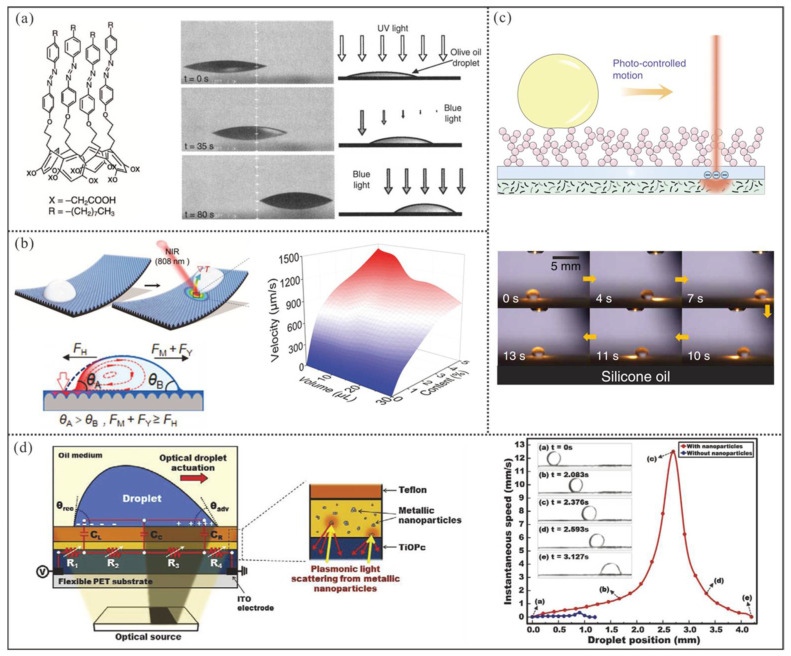
Optical manipulation of droplets: (**a**) Droplet manipulation driven by a light-responsive wettability gradient on a photosensitive material-modified substrate. Reproduced with permission from Ref. [121]. Copyright 2000 SCIENCE. (**b**) Droplet manipulation driven by Marangoni flow on a super-slippery surface based on the photothermal effect. Reproduced with permission from Ref. [126]. Copyright 2018 WILEY-VCH. (**c**) Droplet manipulation driven by dielectrophoretic force on a superomniphobic surface based on the photothermoelectric effect. Reproduced with permission from Ref. [129]. Copyright 2020 SCIENCE ADVANCES. (**d**) Droplet manipulation driven by electrowetting gradient force on a hydrophobic substrate based on the photoconductive effect. Reproduced with permission from Ref. [133]. Copyright 2020 Elsevier.

In comparison, azobenzene represents a classic photosensitive compound with excellent light responsiveness. In Ichimura’s pioneering work [121] (Figure 11a), olive oil droplets were deposited on a photo-responsive azobenzene-modified surface. Through UV irradiation, the substrate’s surface energy was effectively enhanced. Subsequently, **asymmetric blue light illumination** induced **gradient surface energy recovery**, which creates **a wettability differential** across the droplet that enabled continuous directional motion. Theoretically, this approach enables droplet transport across arbitrary distances on the substrate, where droplet motion can be immediately arrested through uniform blue light illumination. However, the system still exhibits notable limitations: (1) a substantial 20 s switching delay during UV/blue light alteration for wettability transition, and (2) constrained surface energy gradients generated by the photoactive molecules, ultimately restricting the maximum achievable droplet velocity to sub-160 μm/s levels.

#### 4.3.2. Near-Infrared Light-Based Manipulation

Regarding near-infrared light (NIR), the methods for droplet manipulation can be primarily categorized into two major types. The first approach utilizes the photothermal effect to create surface thermal gradients for inducing surface tension gradients at either liquid–liquid interfaces or droplet surfaces, which can induce a wettability gradient or Marangoni flow to actuate the droplet [125,126,127,128]. For instance, Gao et al. [126] incorporated Fe_3_O_4_ nanoparticles into a PDMS-based organogel and fabricated a heat-absorbing PDMS substrate with a nanostructured surface that retained silicone oil (Figure 11b). Herein, the nanostructured surface was achieved through thermal curing on an anodic aluminum oxide (AAO) template, followed by silicone oil swelling and demolding. Under localized NIR irradiation, the surface energy gradient of the silicone oil layer provided a wettability gradient force for droplet transport, while the Marangoni flow inside the droplet contributed to the driving stress. Under the joint effect of these two forces, various droplets can be effectively actuated at speeds below 1.5 mm/s.

In the second approach combing photothermal effects with pyroelectric crystals [118,129,130], the temperature rise from localized optical irradiation can induce a reduction in the spontaneous polarization intensity of pyroelectric crystals. Consequently, additional produced surface free charges can create an electric field to drive droplet transport. For example, Li et al. [129] developed a droplet manipulation platform based on the pyroelectric effect by sandwiching a lithium niobate (LiNbO_3_) crystal between a superomniphobic film and a photothermal film (Figure 11c). The superomniphobic top layer exhibited an ultra-low rolling angle of less than 10° even for silicone oil (18.9 mN m^−1^). When NIR light passed through the semi-transparent superomniphobic surface and pyroelectric crystal to reach the underlying photothermal film, the locally irradiated region experienced a rapid temperature increase. Droplets on the superomniphobic surface were then propelled toward the irradiated spot via dielectrophoretic forces from the localized electric field.

For both methods, the irradiation duration of the platform decreases as the infrared laser spot accelerates, which results in localized temperature reduction. As a result, dynamic thermal gradients or electrostatic charge accumulation on the platform exhibits a corresponding decrease, which ultimately constrains the driving force. Although the second approach demonstrates superior droplet mobility, the maximum actuation velocity remains limited to 5 mm/s. Particularly for low-permittivity silicone oil, the actuation velocity is merely approximately 1 mm/s. Furthermore, the evaporation-induced loss caused by additional infrared heating of droplets requires further mitigation.

#### 4.3.3. Visible Light-Based Manipulation

For visible light, the most widely used approach is to use the visible light-induced electrowetting effect for droplet manipulation [131,132,133]. Under visible light illumination, the substrate beneath a droplet can generate a light-induced local voltage drop, altering the local wettability of the substrate and enabling droplet motion. As shown in Figure 11d, Thio et al. [133] spin-coated a photoconductive layer (titanium oxide phthalocyanine, TiOPc) onto a PET substrate with indium tin oxide (ITO) electrodes at both ends. To enhance the photoconductive layer’s performance, they further applied metallic nanoparticles to exploit their light-scattering effect, followed by a Teflon layer to provide hydrophobicity and dielectric properties. As a DC voltage was applied between the two ITO electrodes at the substrate edges, the TiOPc layer and the dielectric layer function as a series-connected photosensitive resistor and a parallel capacitor, respectively. Herein, the impedance of metal nanoparticles and the droplet is negligible. When projected light asymmetrically illuminates one side of the droplet, the resistance of the TiOPc layer increases in the irradiated region, which leads to a higher voltage drop on that side. Finally, an electrowetting gradient force was generated for droplet manipulation. However, due to the limited ability of light to induce voltage drops, the effective average actuation speed is restricted to below 1.5 mm/s.

### 4.4. Acoustic Manipulation

Acoustic actuation enables droplet manipulation by directly or indirectly converting acoustic energy into kinetic energy of droplets. Based on distinct actuation mechanisms, there are two main categories including acoustic radiation pressure-based [134,135,136,137,138,139,140] and acoustic streaming-based droplet manipulation [141,142].

#### 4.4.1. Acoustic Radiation Pressure-Based Manipulation

**Figure 12:**
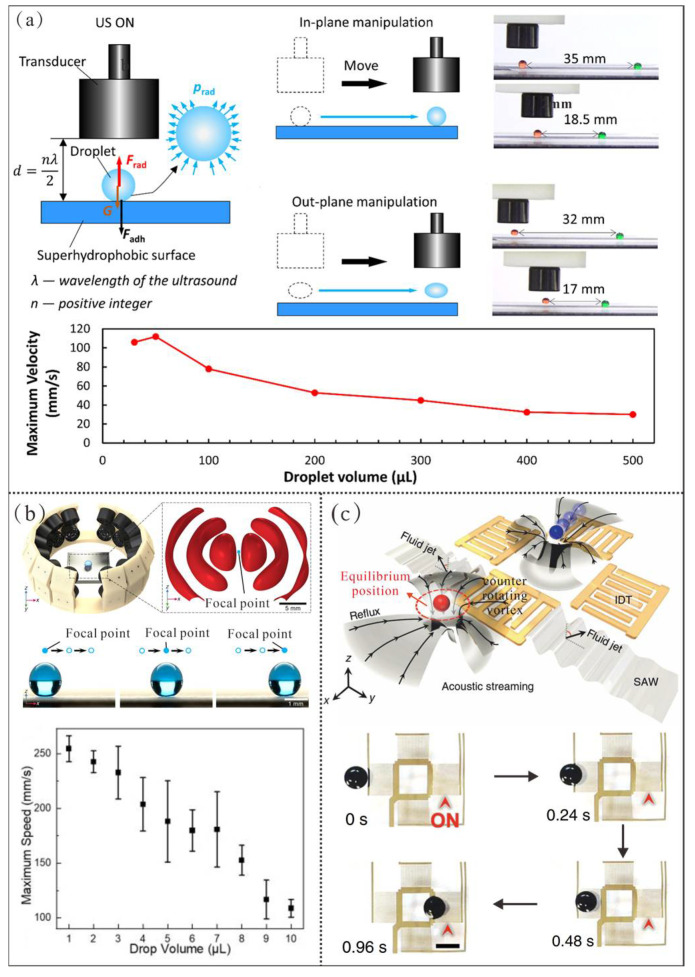
Acoustic manipulation of droplets: (**a**) Droplet manipulation based on mobile ultrasonic standing-wave levitation. Reproduced with permission from Ref. [140]. Copyright 2023 The Royal Society of Chemistry. (**b**) Droplet manipulation using tunable twin-trap foci of an ultrasonic phased array. Reproduced with permission from Ref. [138]. Copyright 2023 SCIENCE ADVANCES. (**c**) Droplet manipulation via a hydrodynamic trap induced by acoustic streaming. Reproduced with permission from Ref. [141]. Copyright 2018 NATURE COMMUNICATIONS.

Considering the direction, acoustic radiation pressure in the method can be further classified into two main categories, which include acoustic radiation pressure perpendicular to the substrate surface and acoustic radiation pressure along the substrate surface. For example, Luo et al. [140] positioned ultrasonic transducers above a superhydrophobic substrate (Figure 12a). The ultrasonic waves reflected by the substrate generated vertically upward acoustic radiation pressure within droplets, which enabled the droplets on the substrate to partially or completely overcome gravity and adhesion forces. Thus, the droplet can move synchronously with the transducers. Herein, the system featured two primary manipulation modes: in-plane and out-of-plane. In the in-plane mode, the applied acoustic radiation force remained smaller than the combined gravitational and adhesive forces, which allows for droplets to move on the superhydrophobic surface while following transducer movement. The out-of-plane mode occurred when the radiation force exceeded the combined opposing forces, causing droplets to lift off the surface during transport. Notably, the in-plane mode achieved a maximum transport speed of 112 mm/s.

Additionally, Yuan et al. [138] arranged a phased array platform with 28 ultrasonic transducers placed on the upper and lower layers of the scaffold symmetrically, where each ultrasonic transducer faces towards the center of the array with ultrasonic intensity adjustable via the control system (Figure 12b). With the activation of the phased array platform, the focal point within the dual-trap ultrasonic field could be freely adjusted by the control software. Here, the droplet at the focal point maintained overall force equilibrium. By progressively altering the focal point in the x and y directions, precise droplet manipulation was achieved with a maximum controllable speed of 250 mm/s.

Although acoustic radiation pressure-based manipulation has realized a high speed of droplet actuation, the droplets with high-frequency oscillations on the substrate are prone to transition from the Cassie state to the Wenzel state, which leads to droplet pinning. Meanwhile, droplets with low surface tension may also experience surface droplet ejection issues induced by high-frequency oscillations. Additionally, the dissipation of acoustic energy within the droplets converts into heat, resulting in unwanted heating problems.

#### 4.4.2. Acoustic Streaming-Based Manipulation

For the acoustic streaming, the hydrodynamic traps can be induced by which to achieve droplet transport [141,142]. For instance, Zhang et al. [141] fabricated interdigital transducers (IDTs) on a LiNbO_3_ substrate to generate acoustic waves, where each array consisting of four IDTs can be regarded as a fundamental unit for droplet manipulation (Figure 12c). As the excitation force of the activated IDT interacts with the overlying inert carrier oil, a butterfly-wing-shaped flow field forms around the IDT. Under the butterfly-wing-shaped flow field, the far-field reflux first pulls the droplet toward the transducer. Then, counter-rotating vortices resist the backflow. Finally, the droplet is pinned at an equilibrium position on one side of the transducer. Therefore, each droplet can be individually transferred from the existing unit to the next by selectively activating the nearest IDT in the adjacent unit, which enables complex manipulation of droplets. In this experiment, the average transport speed of droplets was approximately 6.8 mm/s. Although this method holds great potential for manipulating various types of liquid droplets, the droplet transport capability is still significantly constrained.

### 4.5. Magnetic Manipulation

Magnetic digital microfluidics [143,144,145,146] can be actuated by either permanent magnets or electromagnets, where the incorporated magnetic additives serve as the core actuators enabling droplet manipulation. Depending on whether magnetic elements are added to the droplets, the manipulation strategies can be classified into two categories: magnetic droplet-based [147,148,149,150,151,152,153,154,155,156,157,158] and non-magnetic droplet-based [6,12,159,160,161,162,163,164,165] manipulation.

#### 4.5.1. Magnetic Droplet-Based Manipulation 

**Figure 13:**
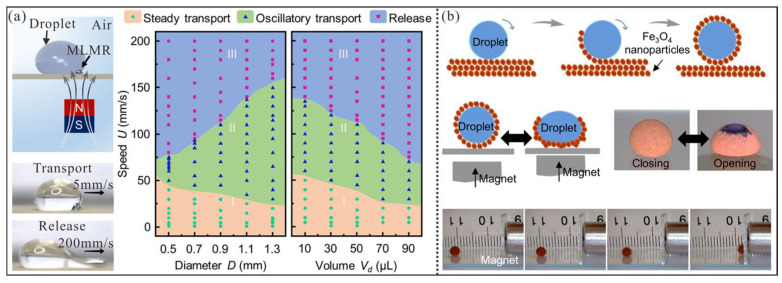
Magnetic manipulation of droplets (**a**) Magnetic manipulation of the droplet contains paramagnetic liquid metal. Reproduced with permission from Ref. [153]. Copyright 2022 American Chemical Society. (**b**) Magnetic manipulation of liquid marbles composed of the droplet encapsulated with hydrophobic Fe_3_O_4_ nanoparticles. Reproduced with permission from Ref. [154]. Copyright 2012 Microfluidics and Nanofluidics.

For droplets containing magnetic elements, the magnetic elements pull the droplet toward the center of the well under the influence of a nearby magnetic potential well. By simply controlling the speed and trajectory of the potential well, the droplet can be directed to move in any desired direction, which enable long-distance droplet transport. For example, Zhang et al. incorporated iron particles into hydrophilic liquid metal to create a magnetic liquid metal robot (MLMR), which is suitable for droplet manipulation (Figure 13a) [153]. Since the shape of the MLMR is determined by the interplay of surface tension, gravity, and magnetic forces, and the shape directly dictates the maximum adhesion force between the robot and the droplet, which is utilized for droplet actuation. Thus, the droplet containing an MLMR was placed on a superhydrophobic surface to allow for shape modulation, which is to acquire the maximum adhesion force for droplet manipulation. In their study, there are three manipulation modes including steady transport, oscillatory transport, and release. Herein, the maximum transport speed (180 mm/s) of water droplets occurred in oscillatory transport mode. However, droplet manipulation results from the combined effects of surface tension, drag force, magnetic forces, and magnetic elements in the system. While increasing the magnetic force or the surface area of magnetic elements can enhance driving force to some extent, low surface tension and velocity-dependent drag make it easier for magnetic particles to escape from the droplet, which leads to failed actuation. Thus, the method is unsuitable for low-surface-tension liquids. Additionally, magnetic elements can be extracted on demand, but residual liquid often adheres to magnetic beads, which raises concerns about cross-contamination.

For droplets with surface-coated magnetic particles, the approach typically involves liquid marbles. For instance, Zhao et al. encapsulated water droplets with a layer of superhydrophobic Fe_3_O_4_ nanoparticles (Figure 13b) [154]. The coated magnetic particles prevented direct contact between the inner water droplet and the substrate, which endowed the droplets with non-wetting properties to achieve the desired magnetic liquid marbles. When a magnet approaches or recedes, these marbles can reversibly open and close. By converting the magnetic attraction between the field and particles into an interfacial force, flexible transport of magnetic liquid marbles is achieved. In Current research, a maximum transport speed of 0.32 m/s was reported. Thanks to their low rolling friction, magnetic liquid marbles exhibit significant potential for high-speed transport. However, shell nanoparticle shedding can contaminate the substrate during transfer and application. Meanwhile, liquid marbles with low surface tension suffer from poor mechanical stability, which hinders long-term reliable operation.

#### 4.5.2. Non-Magnetic Droplet-Based Manipulation

Although magnetic droplet-based manipulation is straightforward and can be moderately adapted for low-surface-tension liquids by adjusting the physical parameters and concentration of incorporated magnetic particles, the resulting contamination issues present significant challenges. Considering this, researchers have begun exploring novel approaches that position magnetic particles externally to the droplet. For magnetic particle-free droplets, manipulation is primarily achieved through magneto-responsive deformation of the wettable surface in contact with the droplet. Depending on the magneto-responsive form of the substrate, it can be mainly categorized into **ferrofluid**-based [12,159] and magnetic elastomer-based [6,160,161,162,163,164,165] magneto-responsive substrates.

**Figure 14:**
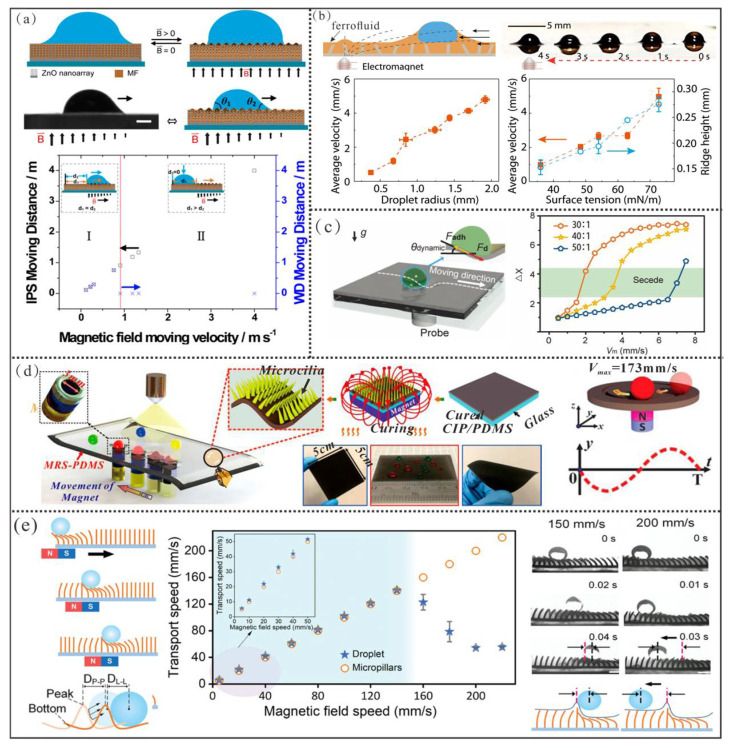
Magnetic manipulation of non-magnetic droplets (**a**) Droplet manipulation driven by wetting gradient forces induced by magneto-responsive roughness on a ferrofluid-infused super-slippery surface. Reproduced with permission from Ref. [159]. Copyright 2016 American Chemical Society. (**b**) Droplet propulsion via net capillary forces induced by magneto-responsive wetting ridges on a ferrofluid-lubricated super-slippery surface. Reproduced with permission from Ref. [12]. Copyright 2021 NATURE COMMUNICATIONS. (**c**) Gravity-driven droplet manipulation using localized dimples on a super-slippery magnetic plate. Reproduced with permission from Ref. [161]. Copyright 2019 WILEY-VCH. (**d**) Gravity-assisted droplet manipulation based on a localized deformation on a superhydrophobic magnetic thin film. Reproduced with permission from Ref. [163]. Copyright 2021 American Chemical Society. (**e**) Droplet transportation enabled by magneto-elastically deformable micropillar arrays generating traveling wave-like actuation. Reproduced with permission from Ref. [165]. Copyright 2022 WILEY-VCH.

For **ferrofluid**-filled super-slippery surfaces, there are two main actuation strategies. The first strategy relies on the roughness gradient formed on the **ferrofluid** surface under a magnetic field, which induces a wettability gradient on the interface for droplet manipulation. For example, Tian et al. infused a ferrofluid into a ZnO nanowire array on a substrate to create a ferrofluid/ZnO nanowire composite interface (Figure 14a) [159]. Under a magnetic field, the ferrofluid self-assembles into microstructures to alter the surface morphology of the composite and influence the wetting state of droplets on it. When a gradient magnetic field is applied, droplets on the composite interface are driven by the wettability gradient force, which achieves a maximum transport speed of 0.9 m/s for water droplets.

**The second strategy** is magneto-responsive meniscus-based droplet manipulation, where a localized magnetic field acts on the ferrofluid meniscus surrounding the droplet, which converts magnetic attraction into a net capillary pull force on both sides of the droplet for directional pumping. For example, Zhang et al. first infused a ferrofluid into a porous surface (Figure 14b) [12]. When a tiny droplet was deposited on this surface, the ferrofluid automatically “wrapped” the droplet due to surface tension, forming a magneto-responsive **“ferrofluid ridge”** around the droplet under capillary forces. With an electromagnet in the experiment activated, the **“ferrofluid ridge”** is drawn by magnetic force, which carries the encapsulated microdroplet with it as they move together toward the magnetic field. In this experiment, the maximum average velocity of the water droplet only reaches 5 mm/s.

Although magnetic particles are no longer directly added to the droplet, the encapsulation by the ferrofluid inevitably introduces contamination of magnetic particles into the droplet, which leads to deviations in reaction or detection results. Furthermore, continuous droplet movement causes gradual loss of magnetic particles, which results in performance degradation or functional failure. Additionally, although magneto-responsive menisci have been proven effective for manipulating liquids with a wide range of surface tensions (25.7 mN/m to 72.8 mN/m), the manipulation speed remains limited to between 1 mm/s and 5 mm/s.

Thus, researchers have begun embedding magnetic elements within elastic substrates. Under moving magnetic fields, the dynamic deformation of magneto-responsive elastomers can be converted into net driving forces for droplet manipulation. Based on the structural configurations of the elastic magneto-responsive substrates, there are three main types including elastic magneto-responsive plates, elastic magneto-responsive thin films, and elastic magneto-responsive micropillar arrays.

**For magneto-responsive elastic plates**, localized magnetic dimples on their surface enable precise droplet manipulation. For instance, Guo et al. developed a magnetic digital microfluidic platform capable of handling various liquid droplets (Figure 14c) [161]. The magneto-responsive plate of the platform consists of three functional layers from top to bottom, which are a lubricant-infused super-slippery surface, a magnetic response layer, and an actuated feedback layer, respectively. Herein, the super-slippery surface minimizes droplet initiation resistance, and the combination of the magneto-responsive and control feedback layers provides sufficient magnetic dimples. Under the influence of a moving magnetic field, droplets within the dimple are driven by their own gravitational component. Although this platform is compatible with most liquid droplets, the driving force provided by magnetic dimples decreases, and the droplet transport resistance on the lubricated surface conversely increases with increasing magnetic field velocity. Consequently, the maximum transport speed is limited to 7.5 mm/s even for high-surface-tension water droplets.

**For magneto-responsive thin films**, their droplet manipulation mechanism is similar to that of magneto-responsive plates. For instance (Figure 14d), Chen et al. first spin-coated a thin layer of magnetic PDMS gel onto a magnetic PDMS film using a magnetic field-assisted template-free process [163]. Under magnetic field exposure, the magnetic particles within the gel self-assembled to form a dense array of micro-cilia, which endows the magnetic PDMS film with superhydrophobic properties. Simultaneously, they developed a magnetic suction nozzle with a circular head. Through initial pressing, this nozzle tightly adheres to the magnetic film to create a localized deformation with sufficiently steep gradients on the superhydrophobic magnetic film surface. As the magneto-responsive deformation moves, the water droplet can achieve a maximum stable manipulation speed of approximately 173 mm/s. This method can manipulate droplets ranging from 2 to 200 μL, which demonstrated capabilities for both anti-gravity and parallel manipulation. Moreover, the surface exhibited excellent mechanical stability to show promise for long-term practical applications. However, it cannot be applied to liquids with surface tension lower than that of water.

**For elastic magneto-responsive micropillar arrays**, continuous droplet transport is achieved through the dynamic interplay between magnetic stress-induced bending and elastic recovery of the micropillars. For instance (Figure 14e), Jing et al. recently employed a template molding method to fabricate an array of elastic magnetic micropillars on a substrate [165]. Subsequently, they further obtained a superhydrophobic micropillar array decorated with nano-sized hydrophobic SiO_2_, which was realized by utilizing the swelling–adhesion characteristics of these micropillars to the hydrophobic SiO_2_ nanoparticles in n-hexane solution. Benefiting from the micropillars’ excellent elasticity, low adhesion, and magneto-responsive properties, the micropillar array generates a depression point and a protrusion point under sufficient magnetic field, whose positions shift synchronously with the magnet’s motion. Here, the depression serves for real-time droplet positioning, while the depression and protrusion act analogously to the bottom and peak of a unidirectional wave. Thus, the deformed micropillars create a traveling wave that propels droplets horizontally. Currently, the maximum stable transport speed for water droplets reaches approximately 150 mm/s. However, liquids with surface tension lower than water may wet the micropillars, rendering them untransportable.

## 5. Conclusions and Perspectives

This review summarizes the latest research progress in bioinspired self-propelled droplet transport and directional droplet manipulation under external stimuli (e.g., electric fields, temperature fields, optical fields, acoustic fields, magnetic fields). The most remarkable feature of bioinspired self-propelled mechanisms lies in their ability to achieve directional droplet movement solely through surface microstructures and chemical properties without external energy input. However, their limitations include relatively slow transport speeds and fixed pathways, which makes it difficult to achieve flexible multidirectional or high-speed movement. In contrast, externally field-assisted droplet manipulation significantly improves flux, speed, and flexibility by leveraging external energy input, yet raises the complexity and cost of excitation system design.

Additionally, electric fields are easily programmable to offer the highest degree of freedom in droplet manipulation, but they are often unsuitable for many organic liquids with low dielectric constants and can cause molecular deposition on substrates to bring contamination. In contrast, thermal manipulation is simpler but typically allows for only one-way transport. Optical fields have distinct advantages in remote manipulation, yet the mechanism relies on converting light into surface energy, temperature fields, or electric fields, which means that the inherent limitations remain unresolved. Acoustic waves enable flexible droplet manipulation through sophisticated acoustic–electric transducer arrays, but high oscillation frequencies render them unsuitable with low-surface-tension droplets. Compared to other methods, magnetic fields stand out due to their excellent penetration and physical/chemical compatibility, demonstrating superior applicability in droplet manipulation. Nevertheless, achieving large-scale parallel droplet control under high-density magnet-controlled unit arrays remain a significant challenge. As shown in Table 1, the main advantages and disadvantages of passive and active droplet manipulation techniques are summarized and compared.

**Figure 15:**
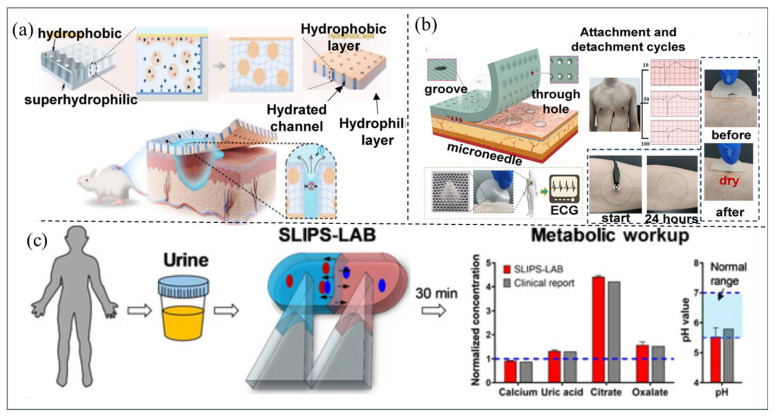
Biomedical applications of microdroplet technology (**a**) Hydrophobic biomimetic surfaces accelerate the healing process of diabetic wounds. Ref. [167]. Copyright 2024 WILEY-VCH. (**b**) A sweat-permeable and multi-mechanism adhesion health monitoring electrode patch. Ref. [168]. Copyright © 2022 American Chemical Society. (**c**) The SLIPS-LAB analysis system is used for rapid detection of analytes related to urinary calculi. Ref. [169]. Copyright 2020 SCIENCE ADVANCES.

It is worth noting that in the biomedical field, with the development of digital microfluidics (DMF) systems, droplet manipulation technology has given rise to various operation methods, such as the manipulation of nanoliter droplets of reagents and cells on electrode array planes [166]. In recent years, in the biomedical applications of microdroplet technology, scholar Sagar N. Agnihotri and his colleagues have developed droplet microfluidic technology and applied it to the field of cancer immunotherapy [17]. Xiao et al. [167] have developed a self-pumping organic hydrogel dressing that can enhance the drainage effect of viscous liquids, thereby accelerating the healing process of diabetic wounds (Figure 15a). Zhang et al. [168] have designed a health monitoring electrode patch with sweat permeability and multi-mechanism adhesion properties (Figure 15b). Additionally, Hui Li et al. [168] have demonstrated a bioanalytical system based on SLIPS-LAB (Super lubricity-Inspired Porous Surface Laboratory) for rapid detection of analytes related to urinary tract stones (Figure 15c).

As mentioned earlier, in the application of droplet manipulation technology, both passive and active methods rely on specific application scenarios due to their inherent mechanisms, advantages, and limitations. For instance, the electrowetting-on-dielectric (EWOD) technology in active methods, with its high precision, suitability for multi-step biochemical detection, and good scalability, is particularly suitable for microfluidic chips. However, it is important to note its dependence on high-voltage power supplies and potential biocontamination issues. In outdoor water collection scenarios, magnetic control technology is more applicable due to its good environmental stability. In thermal transfer applications such as microcooling systems, opto-electrowetting (Opto-EWOD) technology, with its low voltage, low energy consumption, and non-contact control characteristics, is suitable for high-temperature environments, but it has certain limitations in terms of heat dissipation speed. On the other hand, the functional surfaces constructed by passive methods also demonstrate their own advantages. For example, anisotropic microstructured surfaces are suitable for passive efficient water collection; intelligent responsive surfaces are more suitable for microfluidic devices, but it should be noted that when using temperature-sensitive materials (such as PNIPAM), performance failure may occur in extreme temperature or acidic and alkaline environments; isotropic microstructured surfaces, due to their spontaneous wetting characteristics, such as the performance of superhydrophobic surfaces in delaying ice formation, have the potential for further application in thermal transfer and anti-icing fields.

Looking ahead, three major trends are worth noting in the research of droplet manipulation on open planar surfaces of artificial substrates with engineered wettability. Firstly, we should deepen the application of various external-field-based technologies including electrical, thermal, optical, acoustic, and magnetic approaches in droplet manipulation. The focus could be on developing fully automated intelligent control systems capable of autonomous droplet generation and high-speed flexible transport, the purpose of which enable significantly improve the degrees of freedom and efficiency in parallel droplet manipulation.

Secondly, we should investigate synergistic integration pathways between bioinspired self-propulsion (passive) and external field-driven (active) technologies, the aim of which is to leverage their complementary advantages to achieve revolutionary breakthroughs in droplet transport efficiency. Daniel P. Regan and Caitlin Howell developed active control strategies—such as induced temperature gradients, electrical stimulation, and magnetic field exposure—on responsive solid substrates or liquid-covered surfaces to meet the needs of on-demand (PON) diagnostics [170]. Banuprasad TN et al. used temperature-induced wettability gradients to enable rapid, reconfigurable droplet manipulation on PNIPAAm-grafted structured polymer surfaces [22]. Wu D et al. achieved high-performance unidirectional microdroplet manipulation by applying horizontal vibration to a femtosecond laser-fabricated tilted micro-wall array [171]. Jun et al. [172] reported a method for controlling anisotropic wettability using a wrinkled dielectric elastomer actuator. Padinjareveetil et al. [173] fabricated a composite film using femtosecond laser vertical cross-scanning and paraffin injection and integrated it with a silver nanowire heater to create a smart droplet motion control actuator driven by ultra-low voltage. In recent years, Andrea Fergola et al. and Yan Li et al. [174,175] have also reviewed advances in active and passive droplet manipulation technologies.

Thirdly, simplifying and optimizing the liquid compatibility and fabrication processes of wettability-patterned surfaces while improving their durability, the aim of which is to achieve large-scale production to meet the urgent demand for efficient droplet manipulation in practical applications.

## Figures and Tables

**Table 1:** Advantages and disadvantages of active and passive droplet manipulation techniques.

Droplet Manipulation Methods	Energy Consumption	Affected by the Environment	Transmission Speed and Continuity	Requirements for Liquid Working Medium	Manufacturing Cost
**Passive droplet manipulation methods**	**[Advantage]** Droplet directional movement can be achieved merely through surface microstructure and chemical properties, without the need for external energy input.	**[Disadvantage]** Large-scale preparation in an engineering environment is a challenge. **[Disadvantage]** High temperature and high pressure, low temperature, external impact and other factors can all cause damage to the surface of microstructures.	**[Disadvantages]** The transportation speed is relatively slow and the route is fixed, making it difficult to achieve flexible, multi-directional, or high-speed movement.	**[Advantages]** Applicable to most liquid working media. **[Disadvantages]** Not suitable for strongly corrosive solutions or those that damage the surface microstructure.	Moderate cost
**Active droplet manipulation methods**	**[Disadvantage]** There is energy consumption. Among them. **[Disadvantage]** light manipulation can only be converted into surface energy, which has certain limitations.	**[Advantage]** Encapsulating the appropriate external field source can prevent it from being affected by the natural environment. **[Disadvantage]** Electrical manipulation liquid molecules deposit on the substrate, causing contamination.	**[Advantages]** Significantly improved traffic volume, speed and flexibility. Particularly, optical control has an advantage in long-distance control. **[Disadvantage]** Thermal control is relatively simple, but it usually only allows for one-way transmission.	**[Disadvantage]** Electric field manipulation: Not applicable to many organic liquids with low dielectric constants **[Disadvantage]** Acoustic manipulation: High oscillation frequencies make it unsuitable for droplets with low surface tension. **[Disadvantage]** Magnetic manipulation: Large-scale parallel droplet control is difficult to achieve under high-density magnetic control unit arrays.	The complexity of the system design and its relatively high cost.
